# RTF-RCNN: An Architecture for Real-Time Tomato Plant Leaf Diseases Detection in Video Streaming Using Faster-RCNN

**DOI:** 10.3390/bioengineering9100565

**Published:** 2022-10-17

**Authors:** Madallah Alruwaili, Muhammad Hameed Siddiqi, Asfandyar Khan, Mohammad Azad, Abdullah Khan, Saad Alanazi

**Affiliations:** 1 College of Computer and Information Sciences, Jouf University, Sakaka 72388, Saudi Arabia; 2 Institute of Computer Science and Information Technology, Agricultural University, Peshawar 25130, Pakistan

**Keywords:** CNN, Alex net, detection, faster R-CNN, tomato leaf diseases, real-time video streaming

## Abstract

In today’s era, vegetables are considered a very important part of many foods. Even though every individual can harvest their vegetables in the home kitchen garden, in vegetable crops, Tomatoes are the most popular and can be used normally in every kind of food item. Tomato plants get affected by various diseases during their growing season, like many other crops. Normally, in tomato plants, 40–60% may be damaged due to leaf diseases in the field if the cultivators do not focus on control measures. In tomato production, these diseases can bring a great loss. Therefore, a proper mechanism is needed for the detection of these problems. Different techniques were proposed by researchers for detecting these plant diseases and these mechanisms are vector machines, artificial neural networks, and Convolutional Neural Network (CNN) models. In earlier times, a technique was used for detecting diseases called the benchmark feature extraction technique. In this area of study for detecting tomato plant diseases, another model was proposed, which was known as the real-time faster region convolutional neural network (RTF-RCNN) model, using both images and real-time video streaming. For the RTF-RCNN, we used different parameters like precision, accuracy, and recall while comparing them with the Alex net and CNN models. Hence the final result shows that the accuracy of the proposed RTF-RCNN is 97.42%, which is higher than the rate of the Alex net and CNN models, which were respectively 96.32% and 92.21%.

## 1. Introduction

In the current era, agriculture has a vital role in the availability of food. Various crops and vegetable diseases cause a huge amount of loss in yield production [1]. The late blight has a very destructive impact on plant leaves, stems and even fruits in a humid climate [2]. It is very important to protect the plants from blight diseases to improve the crop’s productivity [3]. Effective and proper therapy at the appropriate time is needed to avoid the spread of the disease by early identification [4]. Generally, the experts have provided a manual of care and detection based on their practical experience in recognizing diseases’ symptoms and causes. These specialists must keep a constant eye on the plants in order to prevent disease spread [2]. Continuous monitoring is a tough, time-consuming, and demanding duty for humans, which opens the door to early diagnosis and automation of plant disease detection [3]. For identifying and categorizing plant leaf diseases, several studies have been conducted utilizing various image processing and machine learning approaches [4,5,6].

These approaches used hand-crafted features for the extraction of relevant information from images. Because of their reliance on hand-crafted characteristics, these classifiers lack automation [7]. Furthermore, to reduce the labor of image annotation, small datasets are used for the training and testing of the classifier. In machine learning, using a short dataset is a limiting issue that might lead to overfitting [5,7]. In the recent decade, deep learning (DL) has played a very vital role in the computer vision field and has attracted much research toward itself. Because DL outperforms the state-of-the-art in many domains, various studies have used DL models for different plant disease detection and classification [3]. Various research has developed different classifiers as compared to the traditional machine learning algorithms, which give a more efficient result [2,5,6,8,9,10,11]. Based on its good results, the DL models remain immature and still require more improvement to develop a more practical model for plant disease detection. In the plant disease area, many innovative DL structures have yet to be tried. Based on the accuracy of the results, these DL classifiers are regarded as state-of-the-art models for plant diseases [3,8].

The experienced person and the agriculture expert can evaluate the classification models by sowing its classification procedures. In addition, the agriculture specialist and experienced farmer can use the classifier’s transparency to identify new symptoms that are difficult to detect with the naked eye. Therefore, this paper proposed tomato plant leaf disease detection. Different diseases affect tomatoes in the growing season. Like other crops, tomatoes are also affected by different diseases. Normally, in tomato plants, 40–60% may incur damage due to leaf diseases in the field if the cultivators do not focus on control measures [12]. Three different kinds of diseases, i.e., viral, fungal, and bacterial, are the main cause of tomato plant leaf diseases [13]. With a little computational effort and the accurate detection of leaf diseases to achieve important support, the major techniques of image processing methods are used, which are K-means clustering and segmentation [2]. The virus which is most dangerous is the tomato yellow leaf curl virus (TYLCV) which creates tomato leaf symptoms such as curling upward and yellowing. In tomato production, these diseases can bring a great loss. If these diseases are not identified correctly, then the farmers face a lot of losses. Therefore, we need a proper mechanism for the detection of these problems. As a result, this research demonstrates a deep learning technique and the technological feasibility of using photos and video streaming to assist farmers with the autonomous detection of plant leaves illness and identification. With the use of healthy tomato plant photos, videos, and diseased plant leaves, CNN-based deep faster R-CNN was taught for the identification of various illnesses in the case of tomato plants. In this study for the detection of tomato plant leaf diseases, the three main contributions are presented which are:

- To detect tomato plant leaf disease automatically, a faster R-CNN model is proposed;
- The suggested model uses both images and video to detect tomato plant leaf disease;
- In terms of accuracy, loss, Precision, Recall, and F-Measure, the suggested methodologies are compared to existing models such as Alex net and generic CNN.

The rest of the paper is organized as given. Section 2 will give the related work of this study. While Section 3 explains the model used in this paper. Further, Section 4 discusses the methodology of this paper. Similarly, Section 5 elaborates on the results and discussion of the paper. Finally, last Section 6 gives the conclusion of this research.

## 2. Related Works

It is a very challenging and complex task to develop a reliable and applicable system for a large number of plant diseases’ classifications. For autonomous plant disease classification, most systems have relied on basic feature engineering and machine learning algorithms. For a few classes, all machine learning and feature engineering methods use the same process, where a small change in model architecture and parameters can affect the model performance, which causes less accuracy. Nowadays, CNN has delivered great and impressive results in image classification tasks related to different fields, including agriculture and classification, which allowed researchers to achieve high accuracy in classification problems. Kawasaki et al. [10] proposed a deep CNN model consisting of three convolutional layers, pooling, and the rectified linear unit (ReLU) function are suggested. The paper’s major goal is to use leaf pictures to differentiate healthy cucumbers from sick cucumbers. The datasets utilized in this study included two dangerous viral infections: MYSV (melon yellow spot virus) and ZYMV (zucchini yellow mosaic virus). This collection contains 800 cucumber leaf photos, which comprise (300 with MYSV, 200 with ZYMV, and 300 with no disease). The simulation results reveal that using a four-fold cross-validation technique, the suggested model in this research obtained 94.9 percent accuracy.

Deep CNN was proposed by Sladojevic et al. [13] for plant disease classification using the Stanford background dataset [14]. The dataset utilized in this study is open source and includes 13 types of plant illnesses, one kind of background picture, and one type of healthy leaf. There are 4483 original photos in the collection, all of which are of varying sizes and quality. The simulation reveals that the suggested approach is more accurate in classifying plant diseases. Mohanty et al. [11] used two models such as Alex Net and Google net, for different plant images classification. The dataset was used in this research taken from the public dataset named Plant Village [15], which contains images of 38 labeled classes having 26 diseases of 14 crop species. Three different versions of the dataset are used for the simulation purpose: the first version contains gray-scaled images, the second one contains colored images, and the third one is segmented images to differentiate between the similarities of leaves in the background image. Two standard architectures of the models were used for classification. The configuration results show that a transfer learning model, Google net, had achieved 99.34% of accuracy by distributing colored leaves by 80% of training data and 20% of testing data.

Brahimi et al. [2] applied the CNN-based Alex net and Google net models with transfer learning and fine-tuning mechanisms for tomato leaf disease classifying. In this study, the dataset was taken from the Plant Village public dataset consisting of 14,828 images of nine classes of tomato leaf diseases. This study showed that the accuracy increased from 97.35% to 98.66% by fine-tuning Alex net. Similarly, Google net improves the accuracy from 97.71% to 99.18%. The authors compared the performance of the CNN model against state-of-the-art models like SVM and Random Forest in this study. The accuracy of the shallow models is 94.53 percent for SVM and 95.46 percent for Random Forest, which is less than that of the CNN model. For the categorization of rice illnesses, Lu et al. [5] evaluated the usage of the Alex Net-based CNN model to several machine learning methods, such as SVM, backpropagation (BP), and particle swarm optimization (PSO). With 10-fold cross-validation, the deep model has an accuracy of 95.48 percent, while shallow models like SVM had a 91 percent accuracy, conventional BP had a 92 percent accuracy, and PSO had an 88 percent accuracy, which is considerably superior to the current state of the art models. Liu et al. [16] employed the Alex net model, a deep learning paradigm that extracts CNN features from images and uses these features for classification, and Ferentinos [17] used a deep learning model for the detection and classification of plant disease to obtain superior results. Rangarajan et al. [18] compared the performance of Alex net and VGG19 with a frame size of 13,262 for the detection of tomato crop disease and achieved 97.49% precision. Further, [19] applied transfer learning and the CNN model for accurately detecting infecting dairy crop diseases. The model achieved an accuracy of up to 95% for accurately detecting the infecting dairy crop diseases. Similarly, [20] used the Alex net neural network for tomato leaf condition classification and detection. The models used in this paper achieved an accuracy of 95.75%. Jiang et al. [21] used Resnet-50 to identify different tomato leaf diseases, i.e., with the name of late blight, lesion blight, and the yellow curl leaf. After several iterations, the model predicts the class of diseases with a precision of 98.0% and an accuracy of 98.30%.

Further, [22] used a CNN model with eight layers to detect tomato leaf disease. The tomato leaf dataset from the Plant Village dataset contains different crop datasets using deep learning to achieve a better accuracy rate. The proposed eight-layered CNN techniques show optimal results compared to other classical models [23,24,25,26]. Another study [26] used deep learning methods for tomato leaf disease detection and classification. The author used the different architecture of the CNN-based models with the segmentation technique to achieve better results. The simulation result shows that the proposed CNN-based models achieved better results. Furthermore, in [27], the author used different automatic feature extraction techniques to improve the classification accuracy of the models. To classify and identify tomato disease, this study used the ResNet 50 model. The proposed model in this paper achieved 97% accuracy with different automatic feature extraction techniques. Similarly, [28] proposed an improved VGG-16 for apple leaf disease. The proposed model achieved an accuracy rate of 99.01% for identifying apple leaf disease. Where else in [17] used various CNN architectures for real-time plant disease diagnosis. The authors developed different CNN-based models to construct a real-time system for plant disease detection and classification. In this paper, the author focused on the latest CNN, such as VGG16, VGG19, and ResNet models and their performance. All of the models that have been used in the past have only been used to classify different plant diseases, and only a little amount of effort has been done to identify the afflicted area of the leaf. Also, utilizing multiple CNN-based models, some research has been done for the identification of tomato plant disease. However, the Regional-CNN (R-CNN) model has never been used for tomato plant leaf diseases. As a result of this study, a modified version of R-CNN called Faster R-CNN was developed for the real-time detection of tomato plant disease.

## 3. Materials and Methods

This research is based on region-based CNN. The main processes of R-CNN are discussed in the following subsections.

### 3.1. Region Convolutional Neural Networks (R-CNN)

The Region-CNN consists of the same structure as CNN and is widely used for image classification and detection. The main theme of this paper is image detection using Region-based Convolutional Neural Networks. Where in R-CNN, the R stands for the region; by the region, we mean the desired area which we are interested in detecting. A common CNN can classify an element easily by returning the class name of that object. But cannot show you the exact location of that object in the desired class or image. To characterize bounding box objects known as Regions of Interest (ROI), R-CNN employs a selective search strategy. It then returns the results after extracting the necessary CNN features from each bounding box region [29]. But the R-CNN has some limitations.

- The main limitation of R-CNN is slow training. Its training phase increases if there are more areas or objects to detect or classify;
- Secondly, as it takes a long time to train, the R-CNN cannot be considered a real-time detector as its detection process takes more time for simulation.

In Ref. [30], the author improves R-CNN by naming it fast R-CNN in order to overcome the limitations of the previous model. In the following section, we’ll go over the updated Fast R-CNN.

### 3.2. Fast R-CNN

In [30], the authors overcome different limitations of R-CNN to increase the detection speed of R-CNN with efficiency and the model is named faster R-CNN. It works the same as the R-CNN algorithm [30]. It sends the input picture to the CNN for the convolutional feature map to be created. Then, using the ROI pooling layer, triggered an interesting region and distributed that region into squares to transform into fixed-size using a feature map. The suggested class is then predicted by the SoftMax layer, which is then sent to the fully linked layer [30]. But still, there are many issues that exist in the fast R-CNN model.

- Firstly, it takes the amount of 20 s for every single test image. And it is a slow detection process;
- Secondly, it is still not accurate for real-time data detection;

Similarly, the Fast R-CNN also has some limitations. So, to solve this limitation, another modification is made to fast R-CNN to decrease the time-consuming issues. The following section discusses the new modified version of the fast R-CNN.

### 3.3. Faster R-CNN

The modified version of fast R-CNN is the f. The main goal of the faster R-CNN is detection since it is frequently employed in the detection field. This model was first published in 2015 in the journal Neural Information Processing Systems (NIPS). Its speed is significantly superior to Fast R-CNN and R-CNN, as the name implies. For a real-time dataset process, this model takes 0.2 s. To detect tomato leaves disease from videos in this research, we used faster R-CNN. Faster R-CNN consists of an intricate design because it contains some moving parts [31]. The suggested model will pick the bounding boxes to identify leaf illnesses. The label probability and bounding box will be assigned to each bounding box.

**Figure 1:**
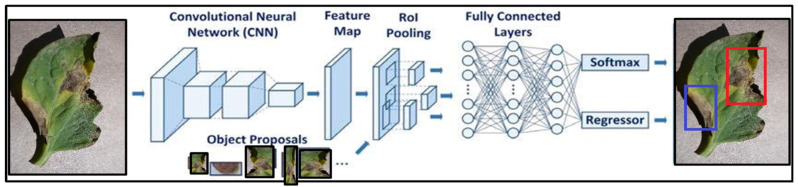
Fast R-CNN architecture.

**Figure 2:**
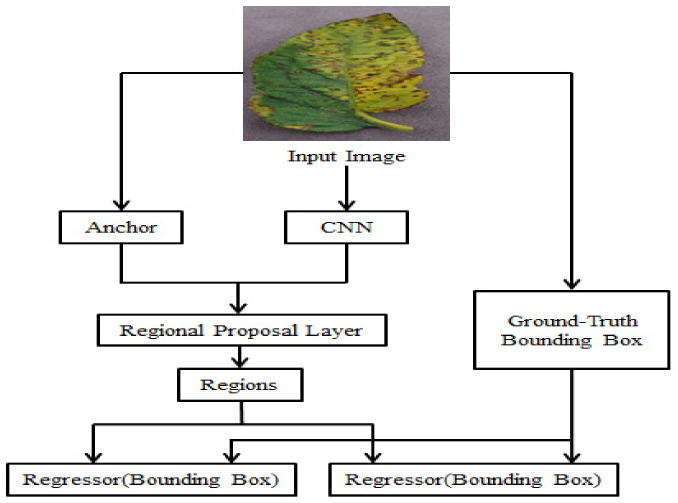
Faster R-CNN network architecture.

The images (multidimensional arrays) are characterized as height × width × depth before going to a middle layer, firstly going over a pre-trained CNN and then ending up with a convolutional feature map. A new ROI pooling layer is introduced in faster R-CNN. A fixed feature-sized vector is obtained from the new ROI pooling layer and is being used to transform the features of maximum pooling within any suggested region validity to a reduced feature map of predefined length. For tomato leaves disease detection, first, the CNN is trained to obtain the feature. And then give this feature to RoI to detect the diseases. The Fast R-CNN architecture is depicted in Figure 1. Figure 2 depicts the improved version of the suggested Faster R-CNN model architecture in the same way. For training, the proposed model used numerous regions of interest (RoI) from input pictures. Each RoI is integrated into a fixed-size featured map, which is subsequently mapped to a feature vector with the use of FC layers. In this network, each RoI has two output vectors: soft-max probabilities and regression offsets of per-class bounding-box bounding.

### 3.4. Proposed Research Framework

**Figure 3:**
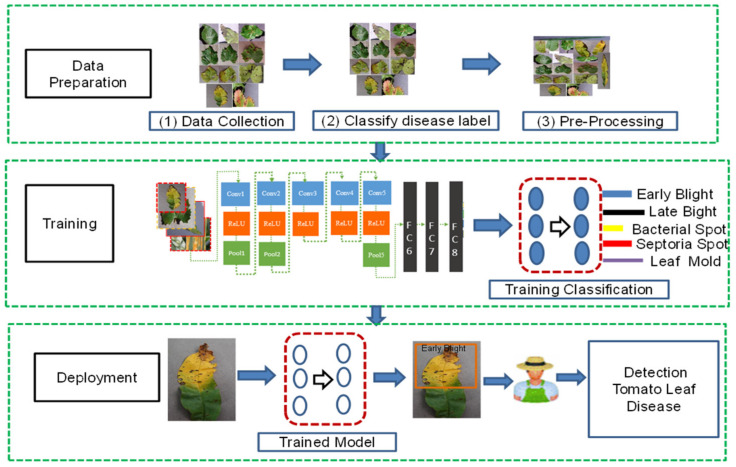
Proposed research framework.

The many steps, such as data collecting, data preprocessing, and suggested model training, are described in this section. The suggested system’s general flowchart is shown below. The step-by-step research framework chart is shown in Figure 3.

## 4. Data Collection

**Figure 4:**
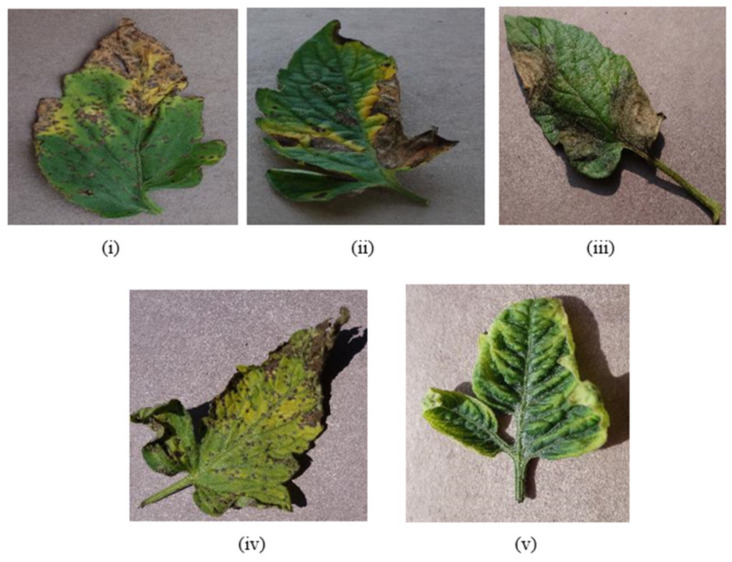
Tomato Leaf Diseases: (**i**) Bacterial Tomato spot (**ii**) Early Tomato Blight (**iii**)Tomato Late Blight (**iv**)Tomato Septoria leaf spot (**v**)Tomato Yellow Leaf Curl Virus.

This research consists of three phases. The first phase is data preprocessing, the second phase is the training phase, and the third phase is the model deployment phase. In this research, the data is collected from the online repository “www.PlantVillage.org” (accessed on 29 October 2018) website, which was recently published in [32]. The dataset which is used in this research is taken from the www.PlantVillage.org website (accessed on 29 October 2018), which is an open-source image repository and consists of more than 50,000 images of leaves. Only the tomato leaves images are extracted in this study. In Table 1, the summary of the dataset is described. This dataset is classified into five different tomato leaf diseases and consists of 12,500 images. The images of infected tomato leaf disease are taken are approximately 100% magnification, and the dimensions of these images are 720 × 1024 pixels which are saved in JPG format. Many images of the dataset are shown in Figure 4.

## 5. Data Preprocessing

In data preprocessing to improve the quality of images or videos, different preprocessing procedures are applied, which are implemented in MATLAB 2018a tool (Natick, MA, US). All the techniques which are used are discussed below.

### 5.1. Resizing Images

To do the categorization, the photos must first be scaled to a specific dimension. The input picture or frame size is identical to the size kept in the database repository. The real size of all the datasets is 960 × 1080 pixels. To resize the tomato leaf images, the following functions are used.

**Figure 5:**
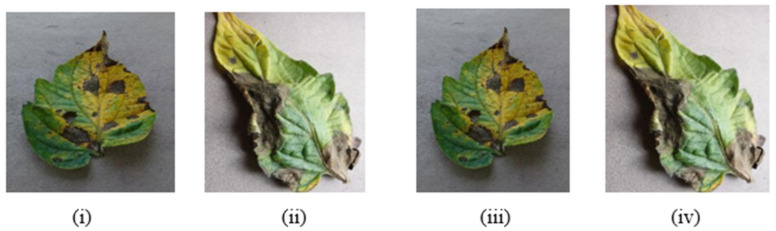
(**i**,**ii**) are the 1080 × 960-pixel of original images, whereas (**iii**,**iv**) are the 256 × 256-pixel reduced images.

X = imresize (I, [numrows numcols])

where X, I, and the numbers are the resultant resized images, the input image, and the number of rows and columns, respectively. The indexed picture, X, is resized with dimension. Imresize delivers a picture that is the same size as the resized indexed image by default. The imresize function’s value pair parameter is used to return a particular dimension that is similar to the original without sacrificing any features. The high image resizing approach is used to shrink images with pixel area relaxation in a variety of studies. The size of the photos is decreased to 256 × 256 dimensions using this technique. Figure 5 displays the original and resized photographs.

### 5.2. Image Enhancement

The next stage is to increase the quality of the photographs once they have been resized. During this phase, 2% of the data must be saturated at low and high concentrations to change the intensity or color map values. For image enhancement, use the following function.

Imadjust (I, stretchlim (I))

**Figure 6:**
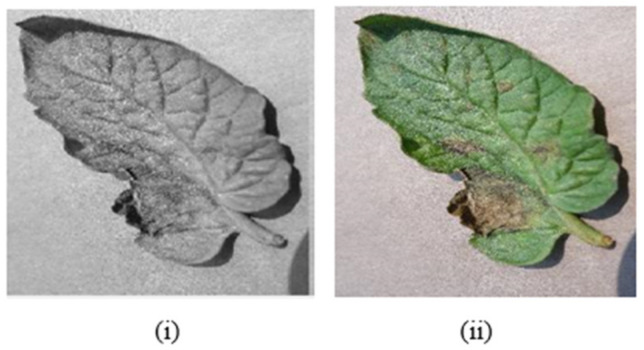
Effect of enhancement before (**i**) and after (**ii**).

The image, I, intensity can be modified to identify a maximum and lower limit, and the above functions return a pixel’s two-element vector. To see how the image was improved, below, Figure 6i depicts the image before enhancement and Figure 6ii depicts the image after enhancement.

### 5.3. Noise Removal

In data preprocessing, the most important step is noise removal in any research. When the frames are extracted from the videos or taken images by the camera, noise may occur in those images. In the case of noise, removal removes the noise as well as some unnecessary data from the photos. The problem of thresholding is simply created by these noise images. So, therefore the removal of noise from frames is most important. The process filters are employed to cancel noise from the photographs in this case. The salt and pepper noise removal method is used to remove noise. The intensity of adjacent pixels and the image background is considerably diverse in salt and pepper noise (sparse light and dark disturbances). Generally, a small number of pixels will be infected by this kind of noise. Dark and white dots can be found in the image. Salt and pepper functions are used in this study to remove noise from the data set. For the removal of noise, we used the following equation.

A = imnoise (I,‘salt & pepper’)

**Figure 7:**
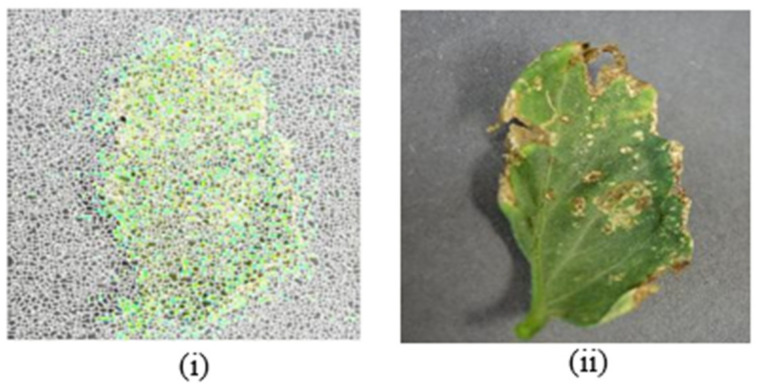
Effect of noise removal before (**i**) and after (**ii**).

With a default noise density of 0.05, the above noise function in MatLab adds salt and pepper noise. As a result, around 5% of the pixels are affected. To add salt and pepper noise to the image, use a noise density of 0.02. The final result is shown in Figure 7.

Figure 7i shows the noise removal images before, while Figure 7ii shows the images after the removal of noise. The image has two qualities once the noise has been removed, one of which is less correlated and has the same feature as the other. As a result, the training process will improve.

### 5.4. Proposed Faster R-CNN

**Figure 8:**
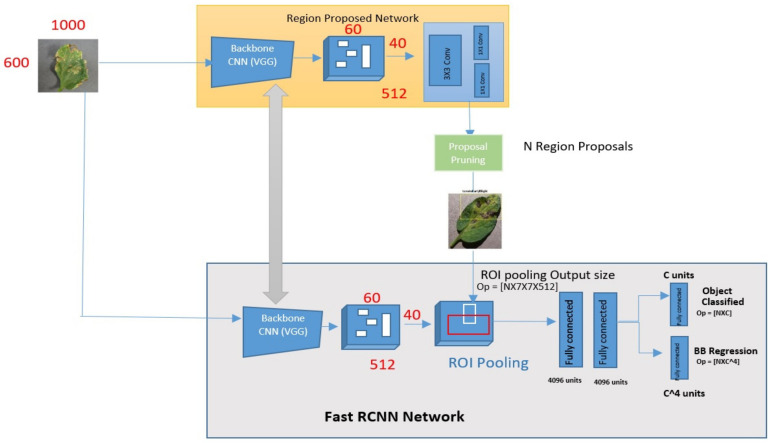
Faster R-CNN detector pipeline.

The modified version of fast R-CNN is called faster R-CNN. This idea was suggested by Ross Girshick in 2015 [31]. R-CNN and fast R-CNN both have problems, and faster R-CNN solves them. The available Faster CNN model has 12 layers and was found to be overfitting. Our proposed model, on the other hand, is a 9-layer augmented R-CNN model. In this architecture, the input image is routed through a CNN to produce a feature map of images. The next step is to complete the RoI pooling. In RoI Pooling, the forward runs shares of a CNN for a picture across all of its sub-regions. Every suggested RoI yields a fixed-sized featured vector in faster R-CNN networks. This is referred to as the ROI pooling layer. The ROI pooling layer converts max pooling features into a fixed-size tiny feature map that may be placed within any valid proposed region. The suggested research consists of simply training a CNN to obtain a feature and then passing these CNN features to RoI for detection. Figure 8 depicts the suggested faster R-CNN architecture. R-CNN is faster since it uses several areas of interest in the input images (RoI). Each RoI is pooled into a fixed-size feature and mapped to a feature vector in fully connected layers (FCs). This network produces two output vectors for each RoI: soft-max probabilities and regression offsets of the per-class bounding box. The input picture is transferred from the backbone CNN first to get the feature map. The bounding box proposals from the backbone feature map are then used to pool the features from the CNN in the RoI pooling layer. As a result, the RoI pooling layer is in place, and it functions as follows:

- Taking the corresponding region from a backbone feature map to a proposal;
- By partitioning the region into a fixed number of sub-images;
- Using max-pooling on sub-windows, you can get a fixed-size output.

The attributes are given into the sibling regression and classification branches through two entirely linked layers. The characteristics are run through a SoftMax layer for each class in the detection to acquire the classification scores and likelihood of each class pertaining to the proposal. The regression layer’s coefficients are used to enhance the projected bounding boxes. The regression is small in this case, but it is unique to each class. As a result, full classes have their own regression with four parameters, each of which corresponds to bounding box output units in the regression layer. The proposed model created in this study is based on Faster R-CNN to recognize and classify tomato leaf disease. The following were included in the suggested model:

Table 2 lists the proposed model’s various parameters, whereas Figure 8 depicts the architecture of the suggested faster R-CNN. In the first step, the suggested model is fed with the data set on tomato leaf disease. The suggested model has nine layers, including convolution layers that are connected to Relu, a fully connected layer that is also connected to Relu, and a fully connected layer that is connected to SoftMax. It also has five classes that classify tomato leaf illnesses. The proposed faster R-CNN model’s is given in Algorithm 1 below as.
**Algorithm 1** Proposed model algorithm *Input: input images/video to the Faster R-CNN model* *Output: display the result of the tomato plant leaf with detected the affected part.* *Start* * Step 1: initialize the structure of the proposed model and initial parameters* * Step 2: Load the input data* * Step 3: Label session of Label Data* * Step 4: Save Session of Label Data* *Step 5: Load Label Session data for Training* *Step 6: Determine the Total number of Images Path in Training* *Step 7: Initialize proposed Faster R_CNN model* *Step 8: read (Size of Label Session)* * For 1 to N* *Calculate error* *For End* *Step 9: For K to Epochs Number Apply in Proposed Method* *CON=>ReLU=>CON* *CON=>ReLU=>POOL* * Set Fully Connected (FC)=> SofMax* * Return Netwrok Architecture Constructed* * For End* *Step 10: Visualization and Process results Post* *End*

## 6. Experiments and Results

The performance of the freshly trained Faster R-CNN is examined in depth in this section, which includes a thorough examination of the convolutional layers as well as the sensitivity of the impacted region.

### 6.1. Preliminaries

The tests were carried out on images of tomato disease leaves collected from [32]. As a simulation tool, MATLAB is employed. The state-of-the-art deep convolutional neural network faster R-CNN was utilized to categorize and detect tomato leaf disease in real-time in this study. The experiment’s dataset was taken from the www.PlantVillage.org (accessed on 29 October 2018) website, which is an open-access photo resource. It has almost 50,000 images of different plant leaves. The dataset for the experiment was obtained from the www.PlantVillage.org website, which is an open-access library of photographs. It has almost 50,000 images of different plant leaves. In this post, only pictures of tomato leaves from this dataset are utilized. The dataset contains 12,500 tomato leaf pictures that are sorted into five categories. There are two sections to the data set for tomato leaf disease: training data and testing data. The Intel Graphical Processing Unit (GPU) has a 16 GB graphics card and 16 GB RAM for research, and it runs Windows 10 with MATLAB R2018a, and the Deep Learning Library installed. For tomato leaf disease detection and classification, we employ an Intel core i3 (CPU) with 8 GB RAM, as well as an HP 720p HD camera (hp, Manufactured by the following: Digilife Technologies, Co., Ltd. 8F, No.51, Ln.258, Ruiguang Rd, Neihu District, Taipei City 114 Taiwan) for real-time testing. In this study, the CNN and Alex net models are respectively shown in Table 3 and Table 4. Also, Table 5 shows the proposed faster R-CNN.

### 6.2. Results

Various trials with accuracy and loss are carried out under all three models. The three models that were employed in the simulation are listed below.

- A faster R-CNN has been proposed;
- Alex net and;
- CNN.

### 6.3. Convolutional Neural Network (CNN) Model

The CNN model’s performance for the categorization of tomato plant leaf diseases is shown in Table 6. On the first iteration, the CNN model had an accuracy of 0.7031 and a loss of 0.5621. The CNN model’s performance improved as the number of iterations increased. The CNN model achieved a 0.9221 accuracy with a 0.465 loss after 30 iterations.

**Figure 9:**
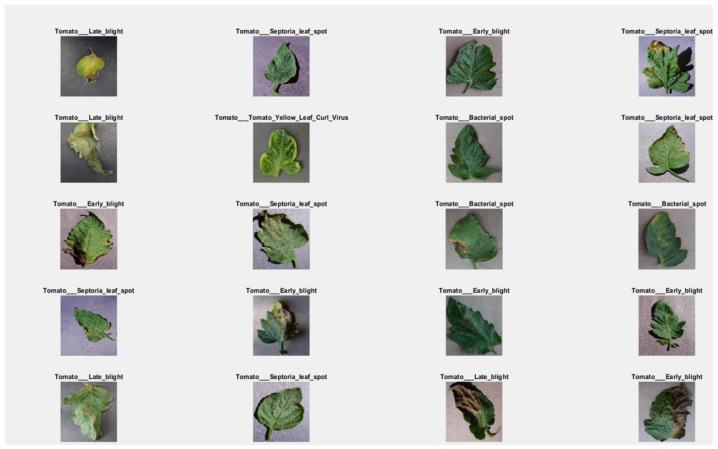
CNN model visual result.

**Figure 10:**
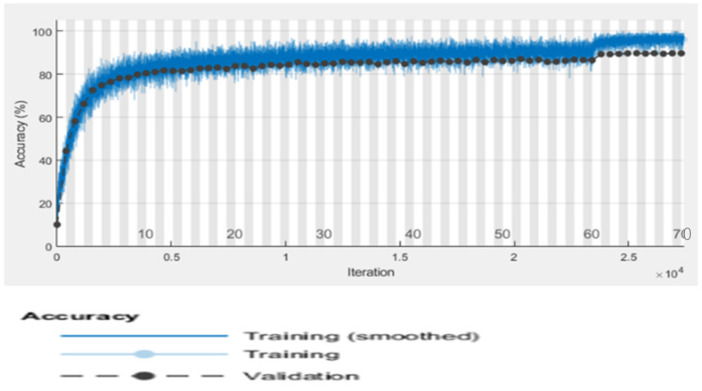
CNN model accuracy.

**Figure 11:**
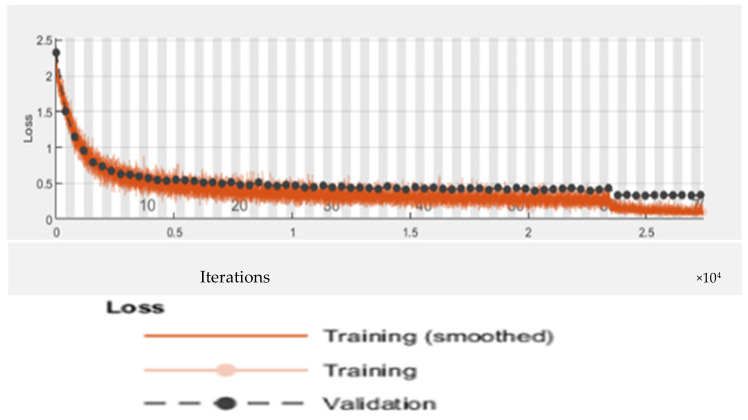
CNN model loss.

Table 7 displays the CNN model’s precision, recall, and f-measure performance on tomato plant leaf diseases at the macro and micro levels. The CNN model had Macro Average precision, recall, and f-measure values of (0.65, 0.69, and 0.66, respectively), while Micro Average precision, recall, and f-measure values of (0.70, 0.71, and 0.70), after training the CNN model, Figure 9 shows different categorization findings on testing data using the illness name as a label. Figure 10 and Figure 11 show the CNN model’s overall accuracy and loss.

### 6.4. Alex Net Model

The Alex net’s proposed model is a modified version of the CNN model. It has eight layers and an input picture size of (227 × 227), containing a Relu (Rectified Linear Unit) and pooling layer to remove non-linearity and down sample the input image to collect more features. Table 8 shows the performance evaluation of the AlexNet model for the tomato plant leaf diseases classification. The Alex net model achieved an accuracy of 0.7531 with a 0.4621 loss on the first iteration, and with various iterations, the performance of the CNN model improved. In 30 iterations, the Alex net model reached 0.9532 percent accuracy with a loss of 0.3065.

**Figure 12:**
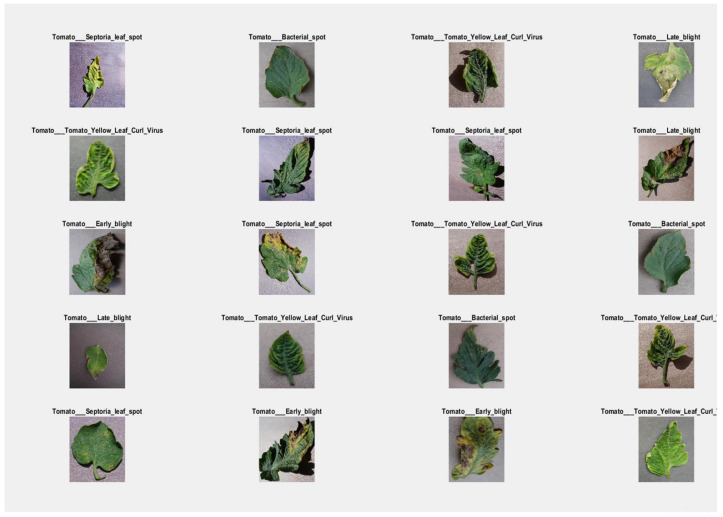
Visual results of Alex Net model testing result.

**Figure 13:**
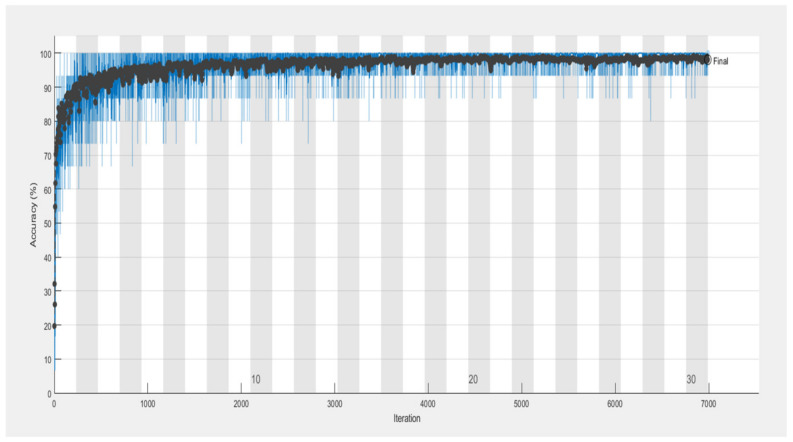
Accuracy of Alex Net model.

**Figure 14:**
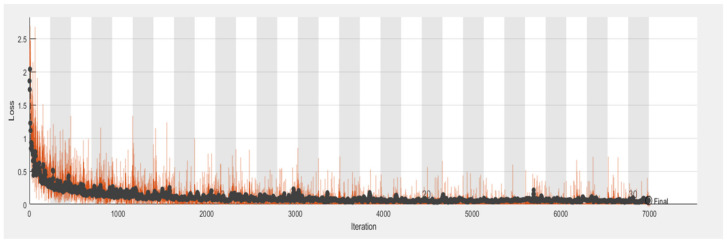
Loss of Alex Net model.

Table 9 displays the Alex Net model’s accuracy, recall, and f-measure performance on tomato plant leaf diseases at the macro and micro levels. The Alex net model has Macro Average precision, recall, and f-measure values of (0.69, 0.74, and 0.71, respectively), and Micro Average precision, recall, and f-measure values of (0.73, 0.76, and 0.74). Figure 12 demonstrates various classification results after training the AlexNet model using illness name as a label on testing data. Figure 13 and Figure 14 show the Alex Net model’s overall accuracy and loss. The black color in Figure 13 show Alex Net model accuracy on testing dataset. Where the blue color show the actual data representation. Similarly in Figure 14 the black color show Alex Net model loss on testing dataset. Where the red color show the actual data representation.

### 6.5. Proposed RTF-RCNN

**Figure 15:**
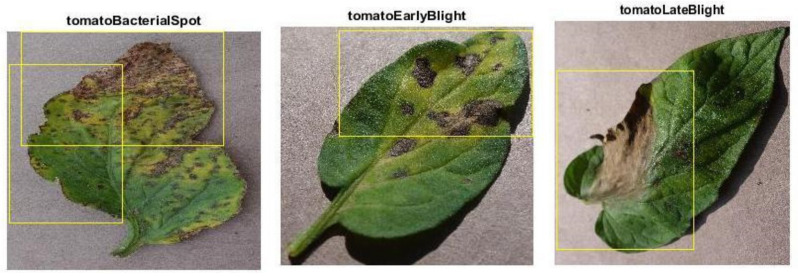

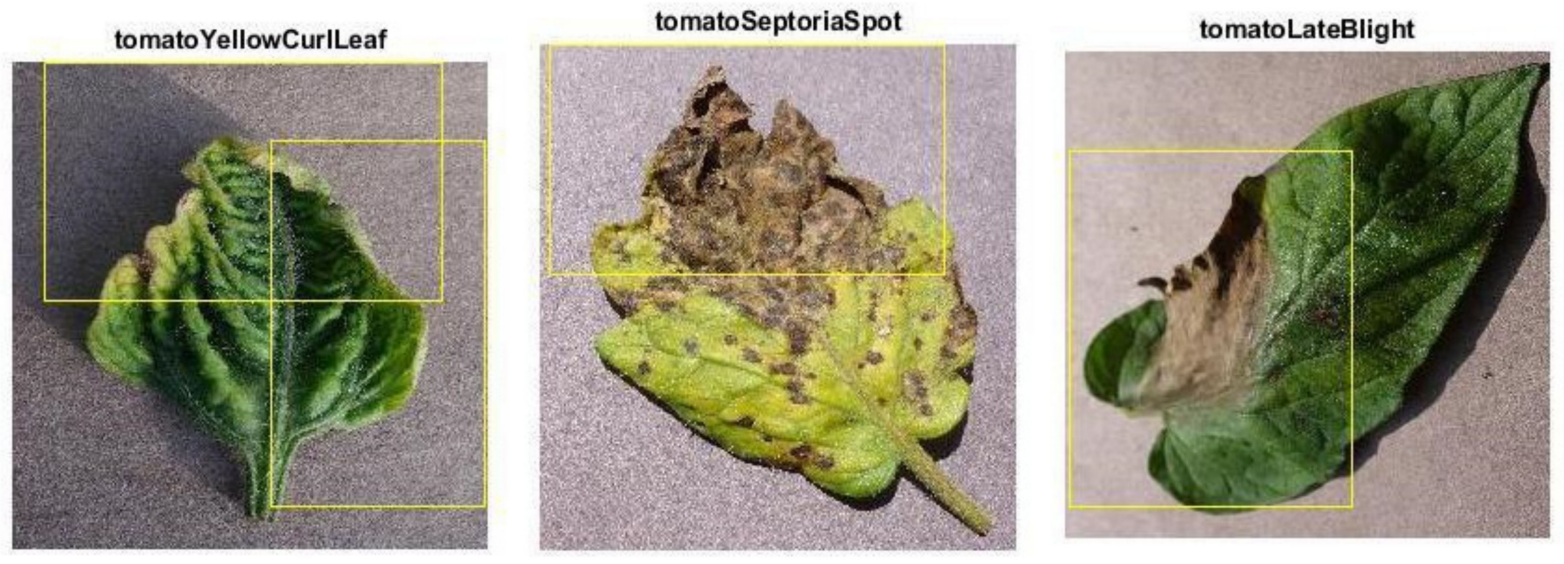
The visual result of the proposed model on testing data.

For the identification and classification of tomato leaves, this study developed a CNN-based model called “faster R-CNN.” The basic faster R-CNN model has 12 layers and has overfitting concerns. In the proposed research study, we made a small modification to the original model by reducing the number of layers from 12 to nine. Thus, we almost get rid of the overfitting issue. In Table 8, the performance evaluation of the proposed model is described. On the first iteration, the proposed model achieved an accuracy of 0.7721 with 0.7631 loss. And the performance is improved after every iteration. The model achieved 0.9742 percent of accuracy with a loss of 0.2765 after 30 iterations. Table 10 show the loss and accuracy performance of the suggested model. Where Table 11 displays the suggested model’s precision, recall, and f-measure performance on tomato plant leaf diseases at the macro and micro levels. The suggested Faster R-CNN model had Macro Average precision, recall, and f-measure values of (0.72, 0.78, and 0.74, respectively), and Micro Average precision, recall, and f-measure values of (0.75, 0.80, and 0.77, respectively). Various detection results of the proposed model with labels are shown in Figure 15. The suggested model can identify and classify different types of tomato leaf diseases, as well as detect diseased areas. In addition, the proposed model operates in real-time. When the suggested quicker R-CNN model is first trained, its accuracy is 76 percent, but after many iterations, the accuracy gradually improves until it reaches 97 percent after 30 iterations.

Figure 15 shows the visual results of several classifications as well as the detection of contaminated areas after training, as well as illness names as a label on the testing data. Like the CNN and Alex net models, the suggested Faster R-CNN model not only classifies but also diagnoses tomato plant leaf diseases. In terms of loss and accuracy, the proposed Faster R-CNN model outperforms the training CNN model and Alex net model, according to simulation results. The entire result demonstrates that the suggested model is capable of detecting tomato leaf disease in real-time with a high degree of accuracy.

### 6.6. Accuracy, Loss, Precision, Recall and F-Measure Comparison Performance

As Table 10 describes, the recommended faster R-CNN model has the greatest accuracy after 30 epochs among the three models, as seen in the three simulated outcomes. Table 12 compares the overall performance of the three models.

**Figure 16:**
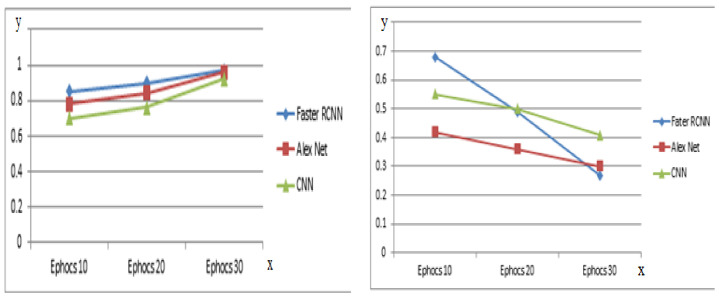
Accuracy and loss Comparison of three Models.

**Figure 17:**
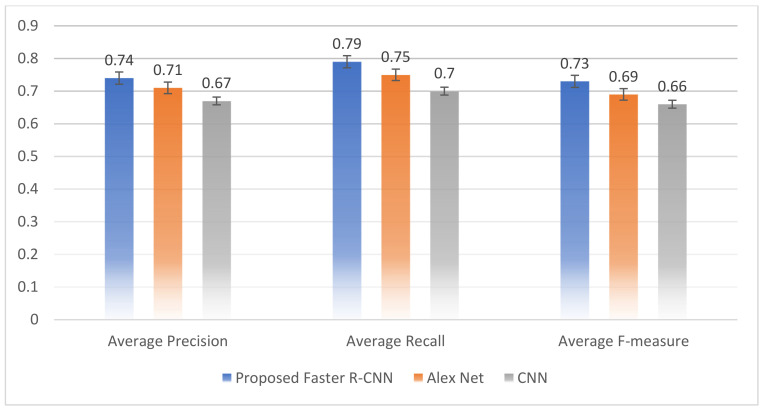
Precision-recall and F-measure performance of three models.

The proposed RTF-RCNN model exhibited the highest accuracy of the three models in 30 epochs, as shown in Table 12. The RTF-RCNN model achieved 97.42 percent accuracy in detecting and classifying tomato leaf disease. While the Alex net model only classified the tomato leaf disease and had a 96.32 percent accuracy rate. Similarly, the CNN model correctly classified tomato leaf disease with 92.21 percent accuracy. The experiment’s findings indicate that the proposed RTF-RCNN model is the most effective at identifying and categorizing tomato leaf disease. Furthermore, Figure 16 also depicts the accuracy and loss comparison between the three models graphically. Similarly, Figure 17 compares the proposed model to the CNN and Alex net models in terms of precision, recall, and f-measure. It is obvious from the graphic that the proposed model outperformed other models in terms of accuracy.

## 7. Conclusions

In the current era, agriculture plays a vital role, and it needs to properly use control measures to improve agriculture production because various crop and vegetable diseases cause a huge amount of loss in plant and vegetable production. Early blight in the plant is a common example of a disease that can damage the crop entirely and can harshly decrease the plant’s production. This research proposed to enhance the existence of Faster R-CNN to detect tomato plant leaf disease and compare different diseases properly. The existence of Faster R-CNN causes the problem of overfitting. To solve this issue, this research elevates the Faster R-CNN for the classification and detection of proper tomato leaf disease. Various studies have been conducted to categorize various plant leaf diseases. However, other CNN-based models were utilized in that study. A small amount of research has been done to classify tomato plant leaf disease using a quicker R-CNN model. This study developed an RTF -RCNN model for the successful detection and classification of tomato plant leaf disease because no one has ever worked on this problem before. A lot of the work has already been done in terms of categorization. This work provided a deep learning-based Faster R-CNN model to increase its performance in detecting and organizing tomato plant leaf illness. The proposed model is compared to contemporary algorithms such as Alex net and CNN in terms of loss, recall, precision, accuracy, and F-measure. The entire simulation shows that the suggested model is superior to other existing models in terms of automatically detecting tomato leaf disease. This proposed model can detect and fix the discovered problem in tomato leaf disease-affected areas, which was a concern in previous models. So, based on simulation findings, this proposed model is approximately the best method in terms of Precision, Loss, Accuracy, and Recall when compared to Alex Net and CNN models.

## Figures and Tables

**Table 1:** Tomato leaf images Dataset Description.

Classes of Tomato Diseases	Number of Images
Yellow Leaf Curl Virus Tomato	2500
Septoria Leaf Spot Tomato	2500
Late Tomato Blight	2500
Bacterial Tomato Spot	2500
Early Tomato Blight	2500

**Table 2:** Details of the proposed model architecture.

Layers	Type	Number Kernel	Kernel Size	Stride
0	Input	3	32 × 32	-
1	Convolution	32	3 × 3	1
2	Relu	-	-	-
3	Convolution	32	3 × 3	1
4	Relu	-	-	-
5	Max pooling	-	3 × 3	2
6	Fully Connected	64	-	-
7	Relu	-	-	-
8	Fully Connected	5	-	-
9	SoftMax	-	-	-

**Table 3:** CNN model structure.

Layers	Type	Number Kernel	Kernel Size	Stride
1	Input	3	32 × 32	-
2	Convolution	32	3 × 3	1
3	Relu	-	-	-
4	Convolution	32	3 × 3	1
5	Relu	-	-	-
6	Convolution	32	3 × 3	1
7	Relu	-	-	-
8	Max pooling	-	3 × 3	2
9	Fully Connected	64	-	-
10	Fully Connected	64	-	-
11	SoftMax	-	-	-
12	Classification	-	-	-

**Table 4:** Parameter of Alex Net.

Layers	Type	Number Kernel	Kernel Size	Stride
1	Input	-	227 × 227	-
2	Convolution	96	3 × 3	1
3	Relu	-	-	-
4	Channel normalization	-	-	-
5	Pooling	-	-	-
6	Convolution	256	5 × 5	1
7	Relu	-	-	-
8	Channel normalization	-	-	-
9	Pooling	-	-	-
10	Convolution	384	3 × 3	1
11	Relu	-	-	-
12	Convolution	384	3 × 3	1
13	Relu	-	-	-
14	Convolution	256	3 × 3	1
15	Relu	-	-	-
16	Pooling	-	-	-.
17	Fully Connected	-	-	-
18	Relu	-	-	-
19	Dropout	-	-	-
21	Fully Connected	-	-	-
21	Relu	-	-	-
22	Dropout	-	-	-
23	Fully Connected	-	-	-
24	SoftMax	-	-	-
25	Classification	-	-	-

**Table 5:** Parameters detail.

Name	Parameters
Algorithm	CNN, Alex net, Faster R-CNN
Convolutional Layers	Relu
Fully Connected Layers	SoftMax
Maximum Number of Epochs	30
Data Set	12,500 images
Training Data	70%
Testing Data	30%
Environment	MATLAB with Deep Learning
Evaluation Parameter	Accuracy, MSE Loss, Precession, Recall and F-Measure

**Table 6:** The performance of CNN Performance at the time of training.

Epochs	Loss	Accuracy	Epochs	Loss	Accuracy
1	0.5621	0.7031	3	0.5571	0.7078
5	0.5501	0.7079	7	0.5490	0.7150
9	0.5431	0.7191	11	0.5521	0.7067
13	0.5771	0.7009	15	0.5921	0.7001
17	0.5831	0.7021	19	0.5710	0.7123
21	0.5322	0.7698	23	0.5201	0.7876
25	0.4901	0.8108	27	0.4690	0.8543
29	0.4321	0.8908	30	0.4165	0.9221

**Table 7:** The average performance of testing data on the CNN Model.

Name	Recall	Precision	F-Measure
Macro	0.69	0.65	0.66
Average	0.66		
Micro	0.71	0.70	0.70
Average	70.50		

**Table 8:** The loss and accuracy of Alex net at the time of training.

Epochs	Loss	Accuracy	Epochs	Loss	Accuracy
1	0.4621	0.7531	3	0.4571	0.7678
5	0.4501	0.7679	7	0.4490	0.7750
9	0.4231	0.7791	11	0.4221	0.7867
13	0.4171	0.8009	15	0.4121	0.8101
17	0.3931	0.8221	19	0.3710	0.8323
21	0.3622	0.8498	23	0.3601	0.8976
25	0.3401	0.9098	27	0.3390	0.9200
29	0.3121	0.9480	30	0.3065	0.9532

**Table 9:** The average performance of testing data of the Alex net model.

Name	Precision	F-Measure	Recall
Macro	0.69	0.71	0.74
Average	0.71		
Micro	0.73	0.74	0.76
Average	0.74		

**Table 10:** Loss and accuracy of the suggested model.

Epoch	Loss	Accuracy	Epoch	Loss	Accuracy
1	0.7721	0.7631	17	0.5731	0.8750
3	0.7571	0.7678	19	0.5210	0.8984
5	0.7401	0.7979	21	0.4922	0.9024
7	0.7090	0.8330	23	0.4401	0.9146
9	0.6991	0.8491	25	0.4001	0.9298
11	0.6891	0.8533	27	0.3590	0.9500
13	0.6371	0.9009	29	0.3121	0.9608
15	0.6221	0.8739	30	0.2765	0.9742

**Table 11:** The average performance of the suggested method on testing data.

Name	Precision	Recall	F-Measure
Macro Average	0.72	0.78	0.74
Micro Average	0.75	0.80	0.77

**Table 12:** The comparison of accuracy, loss, precision, recall and F-measure.

Model	Accuracy	Loss	Precision	Recall	F-Measure
RTF-RCNN	0.9742	0.2765	0.75	0.80	0.77
Alex Net	0.9532	0.3065	0.73	0.76	0.74
CNN	0.9221	0.4165	0.70	0.71	0.70

## Data Availability

Data used for this study and simulation will be provided on demand.
